# Influences of blood lipids on the occurrence and prognosis of hemorrhagic transformation after acute cerebral infarction: a case-control study of 732 patients

**DOI:** 10.1186/s40779-019-0191-z

**Published:** 2019-01-22

**Authors:** Gang Lv, Guo-qiang Wang, Zhen-xi Xia, Hai-xia Wang, Nan Liu, Wei Wei, Yong-hua Huang, Wei-wei Zhang

**Affiliations:** 1 Department of General Surgery, 309 Hospital of Chinese People’s Liberation Army, Beijing, 100091 China; 2 Department of Neurology, Chinese PLA Army General Hospital, Beijing, 100700 China

**Keywords:** Acute cerebral infarction, Hemorrhagic transformation, Total cholesterol, Low-density lipoprotein, Intensive lipid-lowering statins, Anti-platelet, Atrial fibrillation; modified Rankin scale

## Abstract

**Background:**

To study the influence of blood lipid levels on hemorrhagic transformation (HT) and prognosis after acute cerebral infarction (ACI).

**Methods:**

Patients with ACI within 72 h of symptoms onset between January 1st, 2015, and December 31st, 2016, were retrospectively analyzed. Patients were divided into group A (without HT) and group B (HT). The outcomes were assessed after 3 months of disease onset using the modified Rankin Scale (mRS). An mRS score of 0–2 points indicated excellent prognosis, and an mRS score of 3–6 points indicated poor prognosis.

**Results:**

A total of 732 patients conformed to the inclusion criteria, including 628 in group A and 104 in group B. The incidence of HT was 14.2%, and the median onset time was 2 d (interquartile range, 1–7 d). The percentages of patients with large infarct size and cortex involvement in group B were 80.8 and 79.8%, respectively, which were both significantly higher than those in group A (28.7 and 33.4%, respectively). The incidence rate of atrial fibrillation (AF) in group B was significantly higher than that in group A (39.4% vs. 13.9%, *P <* 0.001). The adjusted multivariate analysis results showed that large infarct size, cortex involvement and AF were independent risk factors of HT, while total cholesterol (TC) was a protective factor of HT (OR = 0.359, 95% CI 0.136–0.944, *P* = 0.038). With every 1 mmol/L reduction in normal TC levels, the risk of HT increased by 64.1%. The mortality and morbidity at 3 months in group B (21.2 and 76.7%, respectively) were both significantly higher than those in group A (8.0 and 42.8%, respectively). The adjusted multivariate analysis results showed that large infarct size (*OR* = 12.178, 95% CI 5.390–27.516, *P* < 0.001) was an independent risk factor of long-term unfavorable outcomes, whereas low-density lipoprotein cholesterol (LDL-C) was a protective factor (*OR* = 0.538, 95% CI 0.300–0.964, *P* = 0.037). With every 1 mmol/L reduction in normal LDL-C levels, the risk of an unfavorable outcome increased by 46.2%. Major therapies, including intravenous recombinant human tissue plasminogen activator (rTPA), intensive lipid-lowering statins and anti-platelets, were not significantly related to either HT or long-term, post-ACI poor prognosis.

**Conclusion:**

For patients with large infarct sizes, especially those with cortex involvement, AF, or lower levels of TC, the risk of HT might increase after ACI. The risk of a long-term unfavorable outcome in these patients might increase with a reduction in LDL-C.

## Background

Hemorrhage occurring in the infarction site during acute cerebral infarction (ACI) is called hemorrhagic transformation (HT) [1] and is a complication of ACI. According to the degree of hemorrhage, HT is divided into punctate and patchy hemorrhagic infarction (HI) and space-occupying parenchymal hematoma (PH). HI is further divided into the HI-1 type (small petechiae) and the HI-2 type (more confluent petechiae, without a space-occupying effect), and PH is further divided into the PH-1 type (hematoma < 30% infarct size, mildly space-occupying) and the PH-2 type (hematoma > 30% infarct size, significantly space-occupying) [2]. The incidence of HT, its influences on prognosis, and the risk factors of its formation remain unclear. This study aimed to evaluate the incidence of HT, long-term unfavorable outcome at 3 months after HT, and the risk of HT development to investigate the influence of blood lipids on HT and prognosis after ACI.

## Material and methods

### Study subjects

ACI patients who were continuously admitted and treated in the Department of Neurology of Chinese PLA Army General Hospital between January 1st, 2015 and December 31st, 2016, were retrospectively analyzed. This study was approved by the Medical Ethics Committee of the Chinese PLA Army General Hospital.

### Inclusion criteria

1) Patients who conformed to the diagnostic criteria of ACI [3] and 2) patients with ≤72 h between disease onset and hospital admission.

### Exclusion criteria

1) Patients who had a past history of nervous system impairment caused by stroke and a modified Rankin Scale score (mRS) ≥ 2 points; and 2) patients who had combined liver or kidney damage, blood system diseases, coagulation dysfunction, connective tissue diseases, thyroid dysfunction, tumors, cerebral aneurysm, and cerebrovascular malformation.

### Grouping

Patients were divided into a without HT (A) group and an HT (B) group. Due to limitations in sample size, all types of HT (HI types 1 and 2 and PH types 1 and 2) were included in group B.

### Definition of major indicators

Based on brain magnetic resonance imaging (MRI) or computed tomography (CT) images, the infarct sizes were divided into: 1) large infarct size (infarct lesion > 3 cm^2^ with 2 or more vascular blood supply areas involved); 2) medium infarct size (infarct lesion 1.5~3.0 cm^2^ with 1 vascular branch blood supply area involved); and 3) small (lacunar) infarct size (infarct lesion < 1.5 cm^2^) [4]. HT was confirmed and categorized according to the appearance of abnormally high density on repeated CT films, or hypointensity signals on SWI or GRE sequences of MRI. The second CT was routinely performed within 24 h for patients who received intravenous recombinant human tissue plasminogen activator (rTPA), even if their conditions were stable; for others, the second CT was performed at any time if their symptoms or signs were aggravated. If a patient’s condition was continuously stable, a follow-up CT was ordinarily performed before discharge. If permitted, MRI with SWI or GRE sequences for all patients were performed at the appointed time, which was limited by the number of waiting queues and generally took approximately one week.

Sustained or paroxysmal atrial fibrillation (AF) was confirmed by an urgent ECG when a patient arrived at the emergency department; then, a routine ECG and past history provided by the patient or their relatives were obtained after admission. Further, long-term ECG monitoring was performed for all patients at least in the initial several days until the condition stabilized. A history of hyperlipidemia referred to past hyperlipidemia and continuous use of statins. Blood pressure was measured when the patient was sent to the emergency room or admitted into the hospital. The blood glucose was the random blood glucose result when the patient was sent to the emergency room or admitted into the hospital. The blood lipid and plasma protein levels were the fasting test results in the morning after the patient was admitted into the hospital. Hypoproteinemia (used to evaluate whether a patient was frail or malnourished) referred to both a total protein level lower than 60 g/L and an albumin level lower than 35 g/L. The thrombolysis dose of intravenous recombinant tissue-type plasminogen activator (rTPA) was 0.9 mg/kg. The double anti-platelet therapy consisted of oral administration of aspirin ≥100 mg + clopidogrel hydrogen sulfate tablet 75 mg/d. The intensive lipid-lowering statin (SILL) treatment consisted of an oral administration of atorvastatin ≥40 mg/d.

### Outcome

The outcome after 3 months of ACI was assessed using the mRS: 0 = no symptoms; 1 = symptoms without significant dysfunction; 2 = slight disability and inability to perform all previous activities with ability to look after daily affairs without assistance; 3 = moderate disability requiring some help with ability to walk without assistance; 4 = moderately severe disability and inability to walk or attend to bodily needs without assistance; 5 = severe disability, bedridden, incontinent, and requiring constant care and attention; and 6 = death. Follow-up was completed by physicians in the Department of Neurology whom did not participate in the treatment and was conducted at patients’ subsequent visits or by telephone communication.

### Statistical analyses

Data were analyzed using PASW Statistics (formerly SPSS Statistics) (©Copyright IBM Corporation 2016, IBM Corporation Software Group Route 100 Somers, NY 10589, USA). Quantitative data conforming to a normal distribution are expressed as *χ* ± *s*. Quantitative data that did not conform to a normal distribution are expressed using M(Q_1_-Q_3_). Baseline quantitative data conforming to a normal distribution and homogeneity of variance were examined using the analysis of variance (ANOVA); otherwise, the Wilcoxon rank sum test was performed. The comparison of baseline qualitative data between groups was performed using the *χ*^2^ test or the rank sum test. For analyses of the risk factors of HT and the factors influencing poor ACI prognosis, the raw data were converted into categorical variables (multicategory variables were grouped based on dummy variables) for nonconditional binary logistic regression analyses using the ENTER method. *P* < 0.05 indicated that the difference had statistical significance.

## Results

### Data sets assessed during the study interval

**Fig. 1:**
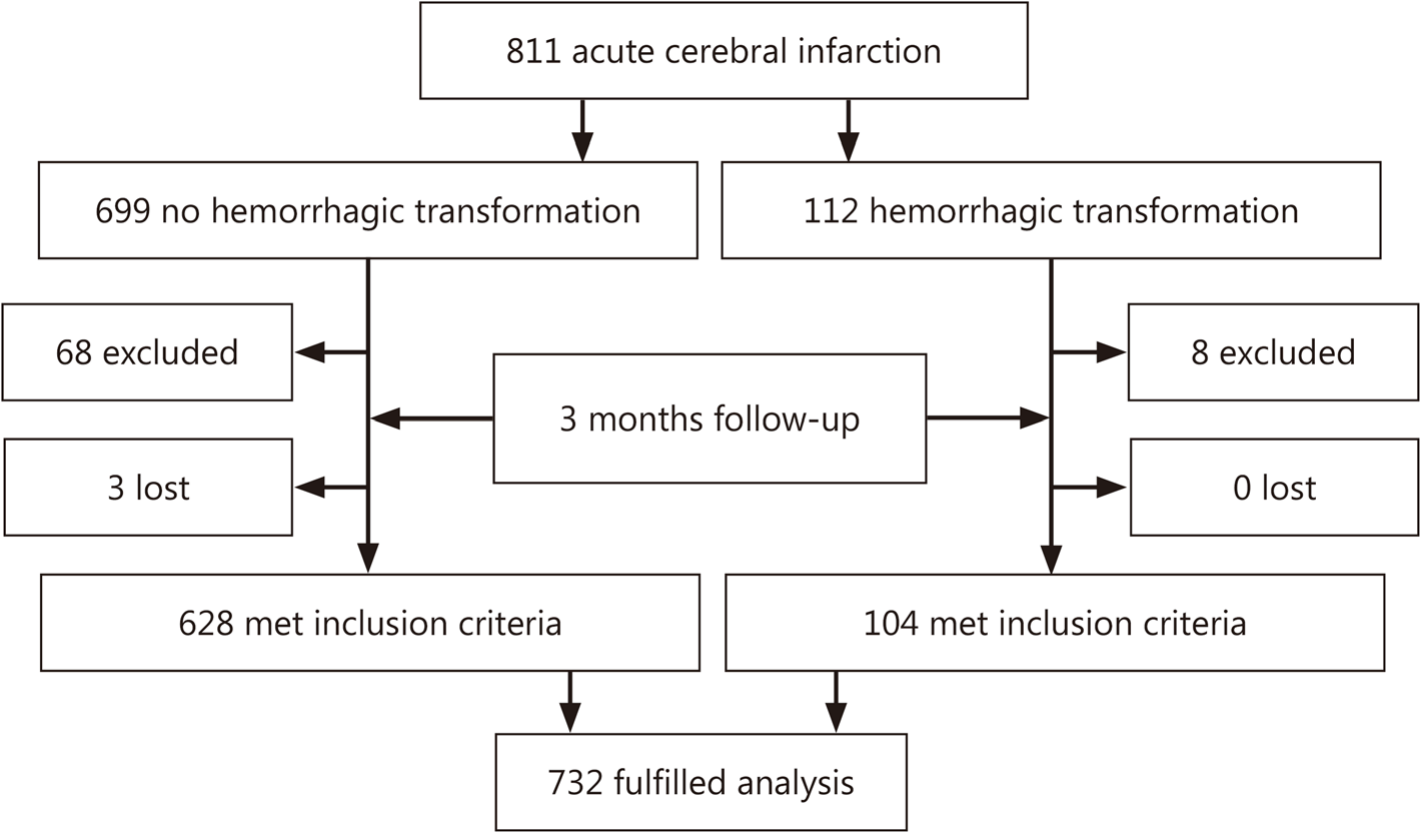
Trial profile

A total of 811 ACI patients were continuously enrolled over 2 years. After enrollment, 76 patients who did not conform to the criteria were excluded (combined with tumors, liver or kidney insufficiency, intracranial infection, intracranial aneurysm, blood system diseases, hypothyroidism, and loss of follow-up at 3 months). A total of 732 patients were enrolled, including 628 in group A (no-HT) and 104 in group B (HT, Fig. 1).

### Baseline characteristics

The age, gender, history of hypertension, history of diabetes mellitus and hyperlipidemia, and blood pressure at admission between these two groups were not significantly different. The overall incidence of AF was 17.5%, and the incidence of AF in group A was significantly lower than that in group B (13.9% vs. 39.4%, *P* < 0.001). The blood glucose in group B was significantly higher than that in group A. TC and TG in group B were significantly lower than those in group A. The percentage of cases with low LDL-C in group B was significantly higher than that in group A. High-density lipoprotein cholesterol (HDL-C) levels and hypoproteinemia were not significantly different between these two groups (Table 1).

**Table 1:** Clinical baseline characteristics

Characteristics	Total (*n* = 732)	Group A (*n* = 628)	Group B (*n* = 104)	*P*
Age, mean ± SD	66.17 ± 13.19	65.81 ± 13.14	68.34 ± 13.34	0.070
< 50	78 (10.7)	69 (11.0)	9 (8.7)	0.697
50–70	368 (50.3)	322 (51.3)	46 (44.2)	0.561
> 70	286 (39.0)	237 (37.7)	49 (47.1)	0.661
Female/Male [*n* (%)]^*X*^	253/479 (34.6/65.4)	211/417 (33.6/66.4)	42/62 (40.4/59.6)	0.178
HBP, no/yes [*n* (%)]	218/514 (29.8/70.2)	190/438 (30.3/69.7)	28/76 (26.9/73.1)	0.491
DM, no/yes [*n* (%)]^*H*^	487/245 (66.5/33.5)	424/204 (67.5/32.5)	63/41 (60.6/39.4)	0.165
Hlip, no/yes [*n* (%)]^*H*^	584/148 (79.8/20.2)	496/132 (79.0/21.0)	88/16 (84.6/15.4)	0.185
AF, no/yes [*n* (%)]^*H*^	604/128 (82.5/17.5)	541/ 87 (86.1/13.9)	63/41 (60.6/39.4)	< 0.001
SBP (mmHg)	150.35 ± 23.26	150.54 ± 23.45	149.20 ± 22.14	0.586
DBP (mmHg)	84.71 ± 13.49	84.83 ± 13.73	83.98 ± 12.00	0.550
NIHSS	7.05 ± 6.27	6.50 ± 6.10	10.39 ± 6.25	< 0.001
Hypoproteinemia, no/yes [*n* (%)]^*H*^	638/94 (87.2/12.8)	550/78 (87.6/12.4)	88/16(84.6/15.4)	0.403
Glucose (mmol/L)	7.33 ± 3.63	7.19 ± 3.57	8.16 ± 3.87	0.011
TC (mmol/L)	4.36 ± 1.05	4.39 ± 1.04	4.15 ± 1.08	0.035
< 3.10	77 (10.5)	58 (9.3)	19 (18.3)	0.857
3.10–5.70	578 (79.0)	507 (80.7)	71 (68.2)	0.047
> 5.70	77 (10.5)	63 (10.0)	14 (13.5)	0.038
TG (mmol/L)	1.41 ± 0.87	1.44 ± 0.90	1.24 ± 0.70	0.030
< 0.57	47 (6.4)	40 (6.4)	7 (6.7)	0.999
0.57–1.7	514 (70.2)	435 (69.2)	79 (76.0)	0.044
> 1.7	171 (23.4)	153 (24.4)	18 (17.3)	0.417
HDL-C (mmol/L)	1.16 ± 0.33	1.15 ± 0.33	1.21 ± 0.32	0.074
< 0.83^*H*^	102 (13.9)	92 (14.6)	10 (9.6)	0.192
0.83–1.96^*H*^	614 (83.9)	523 (83.3)	91 (87.5)	0.184
> 1.96^*H*^	16 (2.2)	13 (2.1)	3 (2.9)	0.106
LDL-C (mmol/L)	2.83 ± 0.94	2.86 ± 0.92	2.67 ± 1.01	0.056
< 2.07	152 (20.8)	124 (19.7)	28 (26.9)	0.001
2.07–3.1	330 (45.0)	285 (45.4)	45 (43.3)	0.443
> 3.1	250 (34.2)	219 (34.9)	31 (29.8)	0.761

^(X)^*. P* values were calculated by one-way ANOVA, Pearson’s Chi-square test; ^*(H)*^. *P* values were calculated by the Kruskal-Wallis *H* ^*(H)*^ test; *HT* Hemorrhagic transformation postacute cerebral infarction; HBP: History of hypertension; DM: Diabetes mellitus; *Hlip* Hyperlipidemia; *SBP* Systolic pressure; *DBP* Diastolic pressure; *NIHSS* National Institutes of Health Stroke Scale; *TC* Total cholesterol; *TG* Triglyceride; *HDL-C* High-density lipoprotein cholesterol; *LDL-C* Low-density lipoprotein cholesterol

### Site and size of ACI

More than 2/3 of infarction sites were distributed in the anterior circulation. The percentages of the large, medium, and small infarct sizes were 15.2%, 20.9%, and 63.9%, respectively. The incidence of HT was 14.2% (104/732). The onset time was 0–26 d, with a mean of 2 d (1–7 d). The percentages of HT corresponding to large, middle, and small infarct sizes were 37.8% (42/111), 27.5% (42/153), and 4.3% (20/468), respectively. HT incidence in anterior circulation infarctions was significantly higher than that in vertebrobasilar artery system infarctions (89/526 = 16.9% vs. 15/206 = 7.3%). The percentages of large and medium infarct sizes and cortex involvement in group B were 80.8% and 79.8%, respectively, which were both significantly higher than the corresponding percentages of 28.7% and 33.4% in group A (Table 2).

**Table 2:** Infarction site [*n* (%)]

Infarction		Total	Group A (*n* = 628)	Group B (*n* = 104)	*P*
Site	ICAs	526 (71.9)	437 (69.6)	89 (85.6)	0.001
	VBs	206 (28.1)	191 (30.4)	15 (14.4)	
Size	0	468 (63.9)	448 (71.3)	20 (19.2)	< 0.001^*a*^
	1	153 (20.9)	111 (17.7)	42 (40.4)	0.074^*b*^
	2	111 (15.2)	69 (11.0)	42 (40.4)	< 0.001^*c*^
Cortex	No	439 (60.0)	418 (66.6)	21 (20.2)	< 0.001
	Yes	293 (40.0)	210 (33.4)	83 (79.8)	

Group A: Acute cerebral infarction without hemorrhagic transformation. Group B: Hemorrhagic transformation postacute cerebral infarction. ICAs: Internal carotid artery system. VBs: Vertebral basilar system. Sites 0, 1, and 2 represent small, medium, and larger areas of infarction, respectively. a. site 0 vs site 1; b: site 1 vs site 2; c: site 0 vs site 2

The percentages of large, medium, and small ACI sizes in AF patents were 37.5%, 34.4%, and 28.1%, respectively, and the corresponding HT incidence rates were 56.1%, 36.6%, and 7.3%, respectively (Table 3). The differences between group A and group B were not significant (Table 3).

**Table 3:** AF-associated infarct sizes and HT

Infarction		Total		Group A [*n* (%)]		Group B [*n* (%)]		*P*
		AF 0	AF 1	AF 0	AF 1	AF 0	AF 1	
Size	0	432 (71.6)	36 (28.1)	415 (76.7)	33 (38.0)	17 (27.0)	3 (7.3)	0.193
	1	109 (18.0)	44 (33.4)	82 (15.2)	29 (33.3)	27 (42.8)	15 (36.6)	0.242
	2	63 (10.4)	48 (37.5)	44 (8.1)	25 (28.7)	19 (30.2)	23 (56.1)	0.056

Size: Area of acute cerebral infarction; *AF* Atrial fibrillation; HT: Hemorrhagic transformation

### AF-associated ACI

Among patients with AF-associated ACT, the percentage of HT with cortex involvement (40.7%) was significantly higher than that without cortex involvement (14.3%). Among patients with sinus arrhythmia-associated ACI, the percentage of HT with cortex involvement (23.2%) was also significantly higher than that without cortex involvement (3.8%) (Table 4).

**Table 4:** HT with cortex involvement in patients with AF-associated cerebral infarction

AF	Cortex	Total	Group A[*n* (%)]	Group B [*n* (%)]	*P value*
0	0	397	382 (96.2)	15 (3.8)	< 0.001
	1	207	159 (76.8)	48 (23.2)	
1	0	42	36 (85.7)	6 (14.3)	0.003
	1	86	51 (59.3)	35 (40.7)	

*AF* Atrial fibrillation. *HT* Hemorrhagic transformation

### National Institutes of Health stroke scale (NIHSS) estimation

Statistical analyses based on the stratification of the degree of the baseline NIHSS showed that HT in patients with NIHSS > 4 points in group B was significantly higher than that in group A (76.0% vs. 46.3%, *P* < 0.001) (Table 5).

**Table 5:** NIHSS and HT

Group	NIHSS [*n* (%)]		*P value*
	≤4	>4	
A (*n* = 628) A (*n* = 104)	337 (53.7) 25 (24.0)	291 (46.3) 79 (76.0)	<0.001

*NIHSS* National Institutes of Health Stroke Scale

### Major treatment

The percentages of patients treated with rTPA venous thrombolysis, argatroban/low-molecular-weight heparin/warfarin anticoagulation, double anti-platelet, and SILL between these two groups were not significantly different. The overall venous thrombolysis rate was 12.8%, and the incidences in group A and group B were 12.4% and 15.4%, respectively (Table 6). The incidence of HT after venous thrombolysis followed by single or double anti-platelet therapy were not significantly different (data not shown).

**Table 6:** Major treatment and prognosis for CH and CH post CI [*n* (%)]

Treatment and prognosis		Group A (*n* = 628)	Group B (*n* = 104)	*P*
rTPA				0.403
	No	550 (87.6)	88 (84.6)	
	Yes	78 (12.4)	16 (15.4)	
Argatroban				0.363
	No	521 (83.0)	90 (86.5)	
	Yes	107 (17.0)	14 (13.5)	
LMWH				0.059^*^
	No	606 (96.5)	96 (92.3)	
	Yes	22 (3.5)	8 (7.7)	
Warfarin				0.536^*^
	No	624 (99.4)	103 (99.0)	
	Yes	4 (0.6)	1 (1.0)	
D-Anti-PLT				0.852
	0, no/single	290 (46.2)	47 (45.2)	
	1, double	338 (53.8)	57 (54.8)	
SILL				0.744
	0, no	443 (70.5)	75 (72.1)	
	1, ILL	185 (29.5)	29 (27.9)	

rTPAvein thrombolysis with recombinant human tissue plasminogen activator. LMWH: Low-molecular-weight heparin. D-anti-PLT (platelets): Aspirin 100 mg or 300 mg + clopidogrel hydrogen sulfate 75 mg; SILL: Intensive lipid-lowering statins. ^*^: Fisher’s exact test (2-sided)

### Analyses of risk factors of HT

Variable assignment (Table 7) for binary logistic regression. Group was the dependent variable, variable with asterisk (*) was the refrence ctaegory.

**Table 7:** Variable assignment

Variables		Assignment
Group	Group (dependent variable)	0 = ACI, 1* = HT post ACI
NIHSS	National Institutes of Health Stroke Scale	0* ≤ 4, 1 > 4
Sex	Gender	0 = female, 1* = male
Age (year)	Age	0* < 50, 1 = 50 to 70, 2 > 70
SBP	Systolic blood pressure	continuous variable
DM	Diabetes mellitus history	0 = no, 1* = yes
Hypopro	hypoproteinemia	0 = no, 1* = yes
AF	Atrial fibrillation history	0 = no, 1* = yes
TC (mmol/L)	Total cholesterol	0* = low, 1 = normal; 2 = high
TG (mmol/L)	Triglyceride	0* = low, 1 = normal; 2 = high
LDL-C (mmol/L)	Low-density lipoprotein cholesterol	0* = low, 1 = normal; 2 = high
HDL-C (mmol/L)	High-density lipoprotein cholesterol	0* < 0.86, 1 = 0.8–1.96; 2 > 1396
Site	Infarction site	0 = Anterior, 1* = posterior circulation
Size	Infarct size	0* = small, 1 = middle, 2 = large
Cortex	Cortex involvement	0* = no, 1 = yes
anti-PLT	Anti-platelet	0 = no*, 1 = aspirin 100 mg or clopidogrel 75 mg; 2 = aspirin 100 mg + clopidogrel 75 mg, 3 = aspirin 300 mg or clopidogrel 300 mg

*. Reference category. ACI (acute cerebral infarction). HT (hemorrhagic transformation post ACI). NIHSS (National Institutes of Health Stroke Scale)

**Fig. 2:**
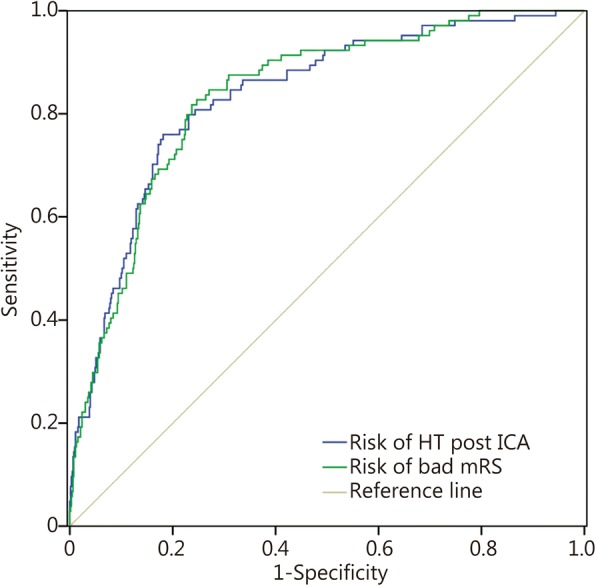
ROC curve

The adjusted logistic regression of the maximum likelihood ratio of the risk factors for HT showed that the model had statistical significance (*χ*^2^ = 143.173, *P* < 0.001). The Hosmer and Lemeshow goodness-of-fit test had a *P* value of 0.985, suggesting that the fit was excellent. The accuracy of prediction was 86.1%. The area under the receiver operating characteristic (ROC) curve was 83.4% (Fig. 2).

After the confounding factors including age, gender, systolic blood pressure, diabetes, and hypoproteinemia were adjusted, the binary logistic analyses of the risk factors of HT showed that infarct size, cortex involvement and AF were independent risk factors of HT after ACI (Table 8). Because there was a clear collinearity between TC and SILL as well as between TC and history of hyperlipidemia with continuous use of statins, SILL and history of hyperlipidemia were not internalized in the regression. The risk of HT in infarctions with large sizes was 7.158 times higher than that in lacunar infarctions and 34.7% higher than that in medium-sized infarctions. The risk of HT in infarctions with cortex involvement was 2.083 times higher than that in deep infarctions. The risk of HT in AF patients was 1.989 times higher than that in patients with sinus rhythm. TC was a protective factor of HT; the lower the TC value was, the higher the risk of HT was. When the normal TC value was reduced by 1 mmol/L, the risk of HT increased by 64.1%. The influences of rTPA venous thrombolysis and anti-PLT on HT were not statistically significant.

**Table 8:** Adjusted logistic analysis for risk factors of HT

Item	B	df	Adjusted *OR*	95% CI		*P value*
				Lower	Upper	
Age		2				0.727
Age (1)	−0.209	1	0.811	0.341	1.930	0.636
Age (2)	−0.006	1	0.994	0.403	2.451	0.989
Sex	0.144	1	1.155	0.674	1.978	0.600
DM	0.476	1	1.609	0.959	2.699	0.072
AF	0.687	1	1.989	1.089	3.632	0.025
SBP	0.000	1	1.000	0.989	1.012	0.937
Hypoproteinemia	-0.201	1	0.818	0.408	1.641	0.572
Site	−0.563	1	0.569	0.297	1.093	0.090
Size		2				0.000
Size(1)	1.670	1	5.310	2.581	10.925	0.000
Size(2)	1.968	1	7.158	3.020	16.962	0.000
Cortex	0.734	1	2.083	1.024	4.236	0.043
TC		2				0.020
TC (1)	−1.025	1	0.359	0.136	0.944	0.038
TC (2)	−0.191	1	0.826	0.223	3.057	0.775
TG		2				0.874
TG (1)	0.264	1	1.303	0.474	3.582	0.609
TG (2)	0.284	1	1.328	0.408	4.329	0.638
HDL		2				0.393
HDL (1)	0.421	1	1.524	0.680	3.418	0.306
HDL (2)	−0.291	1	0.747	0.140	3.993	0.733
LDL		2				0.368
LDL (1)	0.313	1	1.367	0.608	3.075	0.449
LDL (2)	−0.131	1	0.877	0.349	2.207	0.781
Anti-PLT		3				0.058
Anti-PLT (1)	−0.327	1	0.721	0.340	1.528	0.393
Anti-PLT (2)	0.558	1	1.747	0.959	3.182	0.068
Anti-PLT (3)	0.609	1	1.838	0.748	4.520	0.185
rTPA	−0.022	1	0.978	0.492	1.945	0.950
Constant	−3.074	1	0.046			0.012

METHOD = ENTER, Entry = 0.05, Removal = 0.10; Age. 0 < 50, 1 = 50 to 70, 2 > 70 years; Sex. 0 = F, 1 = M; *DM* Diabetes mellitus, *AF* Atrial fibrillation, *SBP* Systolic blood pressure; Site. 0 = Anterior, 1 = posterior circulation; Size. 0 = small, 1 = middle, 2 = large of infarction; Cortex. 0 = untouched, 1 = involved; TC. Total cholesterol, 0 = lower than normal, 1 = normal, 2 = higher than normal; Anti-PLT. Platelets, 0 = no, 1 = aspirin 100 mg or clopidogrel 75 mg, 2 = aspirin 100 mg + clopidogrel 75 mg, 3 = aspirin 300 mg or clopidogrel 300 mg; *TG* Triglyceride, *HDL* High density lipoprotein cholesterol, *LDL* Low density lipoprotein cholesterol, *rTPA* Recombinant human tissue plasminogen activator

### Analyses for outcomes

(Table 9)**:** the overall mortality was 9.8%, and the mortality in group B was significantly higher than that in group A (21.2% vs. 8.0%, *P* < 0.001). The long-term unfavorable outcome rate was 47.5%, and the unfavorable outcome rate in group B was significantly higher than that in group A (76.0% vs. 42.8%, *P* < 0.001).

**Table 9:** Prognosis of the two groups[*n* (%)]

Prognosis		Group A (*n* = 628)	Group B (*n* = 104)	*P*
Died				< 0.001
	No	578 (92.0)	82 (78.8)	
	Yes	50 (8.0)	22 (21.2)	
mRS				< 0.001
	0–2 (good)	359 (57.2)	25 (24.0)	
	> 2 (bad)	269 (42.8)	79 (76.0)	

*mRS* Modified Rankin scale

The adjusted logistic analysis model of the risk factors of a long-term unfavorable outcome was *χ*^2^ = 227.147, *P* < 0.001. The goodness-of-fit test of HT showed *P* = 0.0572. The prediction accuracy was 72.4%, and the area under the ROC curve was 83.6% (Fig. 2).

Large infarct size was an independent risk factor for long-term unfavorable outcomes of neurological functions (Table 10). The risk of bad prognosis in large infarct sizes was 12.178 times higher than that in lacunar infarctions. LDL-C was a protective factor. With every 1 mmol/L of decrease in the normal LDL-C value, the risk of unfavorable outcomes increased by 46.2%. Other confounding factors such as age, gender, AF, systolic blood pressure, diabetes, blood glucose, hypoproteinemia, cortex involvement, TC, TG, anti-PLT and HT had no significant effects on unfavorable outcomes.

**Table 10:** Adjusted logistic analysis of the risk factors of long-term unfavorable outcomes

Item	B	df	Adjusted *OR*	95% CI		*P value*
				Lower	Upper	
Age		2				0.118
Age (1)	0.176	1	1.192	0.660	2.154	0.561
Age (2)	0.524	1	1.689	0.906	3.149	0.099
Sex	−0.064	1	0.938	0.638	1.377	0.743
DM	−0.284	1	0.753	0.515	1.101	0.143
AF	0.228	1	1.256	0.732	2.153	0.408
SBP	0.002	1	1.002	0.994	1.009	0.648
Hypoproteinemia	0.126	1	1.135	0.654	1.970	0.653
Site	−0.030	1	0.970	0.660	1.426	0.877
Size		2				0.000
Size(1)	1.278	1	3.588	2.176	5.915	0.000
Size(2)	2.500	1	12.178	5.390	27.516	0.000
Cortex	0.313	1	1.367	0.862	2.169	0.184
TC		2				0.062
TC (1)	0.701	1	2.016	0.940	4.325	0.072
TC (2)	1.204	1	3.335	1.227	9.065	0.018
TG		2				0.032
TG (1)	−0.392	1	0.676	0.321	1.423	0.302
TG (2)	−0.929	1	0.395	0.170	0.919	0.031
HDL		2				0.985
HDL (1)	0.047	1	1.048	0.606	1.813	0.867
HDL (2)	0.012	1	1.012	0.250	4.098	0.986
LDL		2				0.030
LDL (1)	−0.620	1	0.538	0.300	0.964	0.037
LDL (2)	−0.151	1	0.860	0.456	1.620	0.640
Anti-PLT		3				0.110
Anti-PLT (1)	0.068	1	1.071	0.638	1.797	0.796
Anti-PLT (2)	−0.355	1	0.701	0.452	1.088	0.113
Anti-PLT (3)	−0.554	1	0.575	0.296	1.116	0.102
Group	0.528	1	1.696	0.942	3.054	0.078
Constant	−1.538	1	0.215			0.084

METHOD = ENTER, Entry = 0.05, Removal = 0.10; Age. 0 < 50, 1 = 50 to 70, 2 > 70 years; Sex. 0 = F, 1 = M; Site 0 = anterior, 1 = posterior circulation; Size. 0 = small, 1 = middle, 2 = large of infarction; Cortex. 0 = untouched, 1 = involved; TC. Total cholesterol, 0 = lower than normal, 1 = normal, 2 = higher than normal; Anti-PLT. Platelets 0 = no, 1 = aspirin 100 mg or clopidogrel 75 mg, 2 = aspirin 100 mg + clopidogrel 75 mg, 3 = aspirin 300 mg or clopidogrel 300 mg; Group. 0 = A, no hemorrhagic transformation; 1 = B, hemorrhagic transformation; DM. Diabetes; AF. Atrial fibrillation; SBP. Systolic blood pressure; Hypopro. Hhypoproteinemia; TG. Triglyceride; HDL. High density lipoprotein cholesterol; LDL. Low density lipoprotein cholesterol

## Discussion

The restoration of blood flow perfusion in vessels in ischemic areas after ACI causes different types of secondary HT in different infarct sizes. The CT presentation of HI is a patchy-shaped, high-density image with a blurred border in infarct sites that can be partially fused. Anatomical results by Teal and Pessin [1] have shown that capillaries or veins in the brains of HI patients have multifocal blood outflow. It is generally considered that this type of HI represents erythrocyte exudation through ischemic capillaries rather than vascular rupture. In contrast, PH is hemorrhage resulting from vascular rupture in ischemic areas caused by reperfusion pressure that can have a space-occupying effect or can cause a leak into the ventricle [5]. The presence of these two types of HT can overlap. Animal studies have shown that the function of the blood-brain barrier is very weak. Cerebral ischemia and reperfusion after ischemia can both cause vascular endothelial injury, resulting in further blood-brain barrier leakage [6].

It has been suggested that PH is associated with poor prognosis, while HI is not associated with poor prognosis [7]. Fiorelli et al. [5] found that HI occurring within 36 h of ACI did not have an obvious influence on the long-term outcomes, although HI might suggest that at least some ischemic tissues exhibit reperfusion. However, a single-center retrospective study by Krueger et al. [8] on 299 cases of ACI showed that 87 patients had HT; in addition, PH and HI were both independently associated with unfavorable neurological outcomes. The multivariate analysis results showed that older age, rTPA venous thrombolysis, and vascular recanalization were associated with HT. High NIHSS scores, high blood glucose, proximal middle cerebral artery occlusion, and a history of hypertension at admission increased the risk of HI, suggesting that HI was not a so-called “benign” imaging sign.

Venous thrombolysis at the acute stage of ACI is an effective method for the restoration of blood reperfusion in ischemic areas; however, it may be concurrent with HT or systemic hemorrhage. A retrospective study by Xu et al. [9] on 162 patients showed that 12.3% patients developed HT after rTPA venous thrombolysis. A study by Berger et al. [2] showed that the incidence of HT was 29.5% in the rTPA venous thrombolysis group and 18.5% in the placebo group. The difference between these two groups was mainly because the incidence of PH in the rTPA group was significantly higher than that in the placebo group (7.6% vs. 0.5%, *P* < 0.0001), while the difference in HI between these two groups was not significant. Chen et al. [10] showed that anti-platelet therapy before thrombolysis increased the risks of HT and the 90-d poor prognosis after thrombolysis. A retrospective study by other scholars [11, 12] also showed that anti-platelet pretreatment increased the risk of HT after rTPA venous thrombolysis. The 7-d and 14-d NIHSS scores and the 90-d mRS score in the non-anti-platelet group after rTPA venous thrombolysis were all significantly lower than those in the anti-platelet group after thrombolysis. These results suggested that anti-platelet therapy before and after rTPA could both increase the risk of poor prognosis. Our study showed that the rTPA venous thrombolysis rate was 12.8%, with rates in group A and group B of 12.4 and 15.4%, respectively (*P* = 0.403). This result suggests that rTPA venous thrombolysis did not significantly increase the risk of HT.

A prospective study by Paciaroni et al. [13] that included 4 centers and 1125 cases of ACI showed that the incidence of HT was 8.7%, and the major risk factors of HT included large infarct size, high blood glucose, older age, high NIHSS score at disease onset, and high mean arterial blood pressure. Our study showed that the incidence of HT was 14.2% (104/732); the onset time was 0–26 d, with a mean onset time of 2 d (1, 7); the percentages of infarctions with large, medium, and small sizes were 15.2, 20.9, and 63.9%, respectively; the corresponding incidence rates of HT were 37.8% (42/111), 27.5% (42/153), and 4.3% (20/468), respectively; and the incidence of HT in the anterior circulation area (16.9%) was significantly higher than that in the posterior circulation area (7.3%). A total of 293 enrolled patients in this study had infarctions with cortex involvement, of which 28.3% patients had HT. In group B, the percentages of medium-large areas of infarction and infarctions with cortex involvement were 80.8 and 79.8%, respectively, which were both significantly higher than those in group A (28.7 and 33.4%, respectively). The adjusted multivariate regression analysis results showed that the risk of HT with a large infarct size was 7.158 times higher than that with a small infarct size and 34.7% higher than that with a medium infarct size. The probability of HT in infarctions with cortex involvement was 2.083 times higher than that in deep infarctions. These results indicated that large infarct size and cortex involvement were both important risk factors for HT.

Researches suggested the reasons that large infarctions are prone to HT might be associated with the obvious cerebral edema in infarction lesions and the surroundings that compress peripheral veins to cause blood stagnation and vascular ischemic injury [14, 15]. During ACI, ischemia and hypoxia in the infarct site cause degeneration of the vascular intima and increase the vascular permeability. When edema subsides, and the collateral circulation is established, vascular reperfusion occurs at the infarct sites to cause HT under the impact of blood [1]. Abundant collateral circulation is present in the cerebral cortex; therefore, reperfusion injuries in infarctions with cortex involvement are more severe. Furthermore, animal experiment by Hu et al. [16] found that blood-brain barrier destruction and cerebrovascular self-regulation repair functional impairments caused by ischemia-induced abnormal cellular energy metabolism further induce HT during reperfusion or establishment of the collateral circulation. Infarction in cortical white matter involves the peripheral blood supply areas of the artery; therefore, collateral circulation is usually not sufficient, and lacunar infarction is mainly present. Thus, the incidence of HI is lower; even though HI is present, it mainly includes focal hemorrhage.

The incidence of AF in this study was 17.5%; the incidence in group B was significantly higher than that in group A (39.4% vs. 13.9%, *P* < 0.001). The percentages of large, medium, and small sizes of ACI in AF patients were 37.5%, 34.4%, and 28.1%, respectively, and the corresponding incidence rates of HT were 56.1%, 36.6%, and 7.3%, respectively, which were not significantly different from those in patients with sinus rhythm. However, the incidence of HT in patients with AF-associated ACI with cortex involvement was significantly higher than that in patients without cortex involvement (40.7% vs. 14.3%, *P = 0.003*). The incidence of HT in patients with sinus rhythm-associated ACI with cortex involvement (23.2%) was also significantly higher than that in patients without cortex involvement (3.8%). The adjusted multivariate analysis results showed that AF was another important risk factor for HT.

High blood glucose is also closely associated with HI. High blood glucose causes mitochondrial dysfunction, increases the activity of matrix metalloproteinases, and promotes cell apoptosis to cause HT [17]. Studies by Okamura et al. [18] in a middle cerebral artery ischemia reperfusion model in high blood glucose rats have suggested that high blood glucose promotes enlargement of the infarction lesions, aggravates cerebral edema, and increases the risk of HT through aggravation of energy metabolism disorders. Control of blood glucose by sulfonylureas could significantly reduce HT [19]. The univariate analysis results in this study showed that the percentages of patients with a history of diabetes mellitus were not significantly different between these two groups; however, blood glucose at admission was significantly higher in group B than in group A. The adjusted multivariate analysis results showed that blood glucose at admission did not enter the HT risk factor model (*OR* = 1.609, 95% CI 0.959–2.699) or the long-term poor prognosis model (*OR* = 0.753, 95% CI 0.515–1.101). This result suggests that neither the risk of HT nor bad outcomes after ACI was obviously associated with hyperglycemia.

With the extensive application of lipid-lowering statin drugs, the associations between blood lipids and stroke subtypes has received more attention. The study of Zhang et al. [20] showed that when the TC level increased by 1 mmol/L, the corresponding risk of ischemic stroke increased 25%. One meta-analysis by Zhang et al. [21] of 26 randomized trials about intensive lowering LDL-C including 10 participants showed that with every 1 mmol/L reduction in LDL-C, the annual incidences of heart attack, revascularization, and ischemic stroke were reduced by over a fifth.

Another meta-analysis of 31 randomized controlled trials on statin therapy and the risk of intracerebral hemorrhage by McKinney and Kostis [22] showed that there were no significant differences in the incidence of ICH between the group of 91,588 subjects using statins and the group of 91,215 control subjects (*OR* = 1.08; 95% CI 0.88–1.32; *P* = 0.47). This suggests that the ICH risk was not related to the degree of LDL-C reduction. Moreover, they found that the incidence of total stroke (*OR* = 0.84; 95% CI 0.78–0.91; *P* < 0.0001) and all-cause mortality (*OR* = 0.92;95% CI 0.87–0.96; *P* = 0.0007) were significantly reduced in the statin therapy group. A prospective study by Rocco et al. [23] showed that blood lipid levels and prior statin use were not associated with HT, functional outcomes, or mortality at 3 months. However, Amarenco et al. showed [24] that ACI or transient cerebral ischemic attack treated with atorvastatin (80 mg/d) intensive lipid-lowering therapy increased the risk of HT 66% over that of the control group. The meta-analysis of Wang et al. [25] that included 23 studies showed that TC and cerebral hemorrhage showed a negative level-effect association (with every 1 mmol/L increase in TC, *OR* = 0.85, 95% CI 0.80–0.91). Sturgeon et al. [26] also showed that LDL-C negatively correlated with cerebral hemorrhage. Yang et al. [27] studied 348 cases of ACI and showed that the levels of TG, HDL-C, and LDL-C in 10.1% (35 cases) of patients who developed HT were all lower than those in the control group. A retrospective analysis by D’Amelio et al. [28] on 240 patients with anterior circulation ACI showed that the risk of developing HT in the low TC group (1.95 ± 0.54 mmol/L) was 2.8 times higher than that in the high TC group (2.25 ± 0.48 mmol/L) and that the risk of developing HT in the low LDL-C group (2.66 ± 0.88 mmol/L) was 5.0 times higher than that in the high LDL-C group (3.13 ± 0.97 mmol/L). Olsen et al. [29] found that higher TC levels have been associated with better short-term outcomes after ACI, independently of subtype, vascular territory, age, and glycemia.

The univariate analysis results in this study showed that the TC and TG levels in group B were significantly lower than those in group A and that the percentage of low LDL-C cases in group B was significantly higher than that in group A (26.9% vs. 19.7%, *P* = 0.001). The adjusted multivariate analysis results showed that TC was a protective factor for HT; with every 1 mmol/L reduction in normal TC levels, the risk of HT increased 64.1%. This suggests that low TC levels were associated with HT. Additionally, the adjusted analysis on the risk factors of poor prognosis showed that the risk of poor prognosis was increased in patients with lower levels of LDL-C; for every 1 mmol/L reduction in normal LDL-C levels, the risk of an unfavorable outcome increased 46.2%.

The exact pathogenesis of lipid-related HT after ACI is not yet established. Adequate lipid levels affect the vasculature and are essential for maintaining normal membrane fluidity and the integrity of vessels and their resistance to rupture [30, 31]. Extensively low lipid levels will destroy the integrity of the small vessels and result in blood extravasation over the unstable integrity of endothelial cells in the small cerebral vessels [32]. Bang et al. [33] found low LDL-C levels were independently associated with blood brain barrier permeability derangements, which increased the propensity for hemorrhagic transformation after ACI. An early study by Konishi et al. [34] showed that lower TC resulted in a weakened endothelium that more readily led to arterial fragility, hemorrhage, or slower repair after small hemorrhages. However, this study was based on an association between TC in the range of 3.10–4.65 mmol/L, compared to > 4.65 mmol/L in this study, and on different stroke subtypes, irrespective of cofactors such as age and hypertension.

Our data analysis shows the time of HT onset was mainly within 2 d (1, 7). An explanation for the relationship between 2 d of SILL and the occurrence of HT might be that the dose of statins required by Chinese patients to achieve the same LDL-C target was low, and the Chinese were less tolerant to the same dose of statins [35, 36]. However, all of our patients had history of hyperlipidemia and continuous use of statins prior to the current ACI, and a caveat of our research is that we did not repeatedly test blood lipids, such as at 2 or 7 d after SILL, to compare the changes of all patients’ lipid levels.

A study by D’Amelio et al. [37] showed that the long-term mortality after 3 months of ACI was 12.1% (28/232). The multivariate analysis results showed that large infarct size (*HR* = 2.7, 95% CI 1.2–6.0) and HT (*HR* = 2.3, 95% CI 1.0–5.4) were independent risk factors of long-term poor prognosis. The adjusted multivariate logistic analysis on long-term poor prognosis of ACI showed that large infarct size was an independent risk factor of long-term unfavorable outcomes in neurologic function. The risk of unfavorable outcome in patients with large infarct size was 12.178 times higher than that in patients with small infarct sizes.

## Conclusion

In summary, HT generally occurs 2 d (1, 7) after ACI. Large infarct size, cortex involvement, AF and lower TC are independent risk factors of HT. With every 1 mmol/L reduction in normal TC levels, the risk of HT increases by 64.1%. The risk factors of poor prognosis include large infarct size and lower LDL-C. With every 1 mmol/L reduction in the normal LDL-C level, the risk of unfavorable outcomes increases by 46.2%. Therefore, for ACI patients with AF, large areas of cortex involvement, or low levels of TC, the risk of HT should be vigilantly monitored. Major therapies with intravenous rTPA, intensive lipid-lowering statins and anti-platelets were not significantly related to HT or long-term poor prognosis after ACI.

The limitations of this study are that it is not a prospective case-control study and that the major observation indicator, blood lipids, was measured from fasting blood collected in the morning after admission. In addition, most patients did not receive blood lipid follow-up at the acute stage; therefore, group statistics could not be performed on repeatedly measured blood lipid levels after drug administration.

## Abbreviations

ACI: Acute cerebral infarction
AF: Atrial fibrillation
CT: Computed tomography
HI: Hemorrhagic infarction
HT: Hemorrhagic transformation
LDL-C: Low-density lipoprotein cholesterol
MRI: Magnetic resonance imaging
mRS: Modified Rankin Scale
NIHSS: National Institute of Health Stroke Scale
PH: Parenchymal hematoma
rTPA: Recombinant tissue-type plasminogen activator
SILL: Intensive lipid-lowering statins
